# Primary Aspergillosis

**Published:** 1915-11

**Authors:** 


**Primary Aspergillosis.** G. W. Holden, Med. Rec., page 587, Oct. 2, reports a case, the mould identified as A. fumigatus or nidulans. Patient female, aged 47, apparently tuberculous but no tubercle bacilli found. Dieulafoy first called attention to the possibility of pulmonary aspergillosis in 1890, before which date, it had been found only in the ears, nose and mouth. Henry Sewall, in discussion warns against the possibility of accidental contamination of specimen, as often occurs in urine.

